# Supplementary material for: Toward Standardization of a Lung New Approach Model for Toxicity Testing of Nanomaterials
Source: Nanomaterials (Basel). 2024 Nov 24;14(23):1888. doi: 10.3390/nano14231888 (PMC11643904; doi:10.3390/nano14231888)
Supplement: Supplementary file 1 [file nanomaterials-14-01888-s001.zip › nanomaterials-3262588-supplementary.pdf]

nanomaterials

*Supplementary materials*

# Toward Standardization of a Lung New Approach Model for Toxicity Testing of Nanomaterials

**Elisabeth Elje [1,†], Laura M. A. Camassa [2,†], Sergey Shaposhnikov [3], Kristine Haugen Anmarkrud [2], Øivind Skare [2], Asbjørn M. Nilsen [4], Shan Zienolddiny-Narui [2,*] and Elise Rundén-Pran [1,*]**

[1] Norwegian Institute for Air Research, 2027 Kjeller, Norway; eel@nilu.no
[2] National Institute of Occupational Health in Norway, 0033 Oslo, Norway; laura.camassa@stami.no (L.M.A.C.)
[3] NorGenoTech, 0349 Oslo, Norway
[4] Faculty of Medicine and Health Sciences, Department of Clinical and Molecular Medicine, Norwegian University of Science and Technology; 7491 Trondheim, Norway
* Correspondence: shan.narui@stami.no (S.Z.-N.); erp@nilu.no (E.R.-P.); Tel.: +47-23195284 (S.Z.-N.); +47-63898237 (E.R.-P.)
† These authors contributed equally to this work and shared first authorship.

## 1. Cell cultures

**Table S1.** Cell passages used in experiments on lung triculture models.

| Cell lines | Passages | Laboratory |
|---|---|---|
| A549 | 7, 9, 11, 13, 15 | 1, STAMI |
| | 6, 7, 11, 13 | 2, NILU |
| EA.hy926 | 7, 9, 11, 13, 15 | 1, STAMI |
| | 6, 9, 13, 15 | 2, NILU |
| THP-1 | 6, 8, 10, 12, 14 | 1, STAMI |
| | 7, 12, 16, 21 | 2, NILU |

## 2. Calculation on nominal exposure concentrations

The deposition efficiency in the VITROCELL® system was measured by fluorescein deposition as described in Camassa & Elje et al 2022 [1]. This was used for calculation of nominal concentrations of the NMs according to the following formula[1-3]:

$$C_{nominal} = \frac{C_{sample} \times V_{sample}}{A_{total}} \times DE$$

Nominal concentration = $C_{nominal}$
Concentration of sample = $C_{sample}$
Nebulizing volume of sample = $V_{sample}$ = 800 µL
Total deposition area = Atotal = 145 cm$^2$ [2]
Deposition efficiency = DE = approximately 53.0 %

Calculation of NM-300K exposure concentration:

$$C_{nominal} = \frac{C_{sample} \times V_{sample}}{A_{total}} \times DE = \frac{7000\ \mu g/ml \times 0.8\ ml}{145\ cm^2} \times 0.53 = 20.47\ \mu g/cm^2$$

## 3. Cellular viability and DNA damage

**Table S2.** Cell viability (%) relative to the incubator control (set at 100%) was measured by the alamarBlue assay in Laboratory 1 (Lab1) and Laboratory 2 (Lab2) 24 hours post-exposure to NM-300K (20 µg/cm2) or PBS negative control (PBS 10 % in MQ water) at the air-liquid interface (ALI). The results are presented as the mean ± standard deviation from a total of n=5 (Lab1) and n=4 (Lab2) independent experiments, each with 2 replicate cell culture inserts. The table corresponds to the results in Figure 1. Statistically significant effects compared to the NC (non-exposed control) and PBS (PBS 10% in MQ water) negative control were analyzed by one-way ANOVA with Tukey's multiple comparisons for cells at the apical or basolateral side, with a significance level of a, b, c (apical) or A, B, C (basolateral) at *p<0.05. Here, a, b, c are NC (non-exposed control), PBS 10% in MQ water, NM-300K 20µg/cm2 exposures at the apical side, respectively. A, B, C represent: NC (no exposed control), PBS (exposure control), NM-300K 20µg/cm2 exposures, at the basolateral side. PC stands for positive control; for Lab1, PC was Tryton X-100 lysed cells and for Lab2, chlorpromazine hydrochloride (100 µM).

| | | **Relative Cell Viability (%)** | |
|---|---|---|---|
| | | Triculture | |
| **Cells** | Exposures | Lab1 | Lab2 |
| A549 / dTHP-1 (Apical) | NC | 100 | 100 |
| | PBS 10% in MQ water | 77.6 ± 20.8 | 78.3 ± 10.1 |
| | NM-300K 20µg/cm$^2$ | 40.1 ± 15.4$^{*ab}$ | 82.3 ± 24.2 |
| | PC | 18.4 ± 5.7$^{*abc}$ | 9.4 ± 13.2$^{*a, b, c}$ |
| EA.hy926 (Basolateral) | NC | 100 | 100 |
| | PBS 10% in MQ water | 76.7 ± 7.4$^{*A}$ | 75.8 ± 20.4 |
| | NM-300K 20µg/cm$^2$ | 52.3 ± 20$^{*A, B}$ | 93.2 ± 54 |
| | PC | 21.3 ± 4.6$^{*A, B, C}$ | 4.62 ± 6.5$^{*A, B, C}$ |

**Table S3.** % DNA in tail measured by comet assay in Laboratory 1 (Lab1) and Laboratory 2 (Lab2). SBs indicated DNA strand breaks and SBs + Fpg oxidized or alkylated DNA lesion determined by the formamidopyrimidine DNA glycosylase (Fpg) enzyme-modified of apical (A549 / dTHP1) and basolateral (EA.hy926) cells of lung tricultures cells model. Analysis was performed 24 hours post-exposure to aerosolized PBS 10 % in MQ water and NM-300K 20 µg/cm$^2$. Results are in this table represented as mean ± standard deviation of n=4 independent experiments and n=2 replicates for exposure for Lab1 and n=4 (except for NM-300K EA.hy926 where n=3) independent experiments for Lab2. The table corresponds to the results in figure 2. Statistically significant effects compared to the PBS control were analyzed by one-way ANOVA with Tukey's multiple comparisons for cells at the apical or basolateral side, with a significance of $^{a, b, c \text{ (apical) or } A, B, C \text{ (basolateral)}}$ p<0.05. Internal controls, TK 6 cells for Lab1 and A549 for Lab 2 with and without (reference) Ro 19-8022 and light exposures, were analyzed by one-way ANOVA with Tukey's multiple comparisons, with a significance of p<0.05. a, b, c are respectively: NC (no exposed control), PBS 10% in MQ water (internal control), NM-300K 20 µg/cm$^2$ exposures at the apical side. A, B, C are respectively NC (non- exposed control), PBS 10 % in MQ water (internal control), NM-300K 20 µg/cm$^2$ exposures at the basolateral side. All: all the groups.

| | | **% DNA in tail** | | | |
|---|---|---|---|---|---|
| | | Triculture | | | |
| **Cells** | Exposures | Lab1 | | Lab2 | |
| | | SBs $^{(a, b, c)}$ | SBs+Fpg $^{(A, B, C)}$ | SBs $^{(a, b, c)}$ | SBs+Fpg $^{(A, B, C)}$ |
| A549 / dTHP-1 (Apical) | NC | 6.7 ± 3.1 | 18.5 ± 8.4 | 3.4 ± 1.2 | 6.2 ± 1.6 |
| | PBS 10% in MQ water | 10.9 ± 3 | 19.4 ± 8.3 | 5.2 ± 1.9 | 6.9 ± 3.1 |
| | NM-300K 20µg/cm$^2$ | 12.9 ± 6.2 | 28 ± 8.4 | 9.7 ± 6.8 | 12.5 ± 8.4 |
| EA.hy926 (Basolateral) | NC | 12.8 ± 5.5 | 25 ± 11.5 | 12.1 ± 2.7 | 10.5 ± 2.8 |
| | PBS 10% in MQ water | 17.3 ± 8.8 | 28.3 ± 3.8 | 12.8 ± 1.9 | 12.7 ± 4.4 |
| | NM-300K 20µg/cm$^2$ | 18.6 ± 4 | 38.5 ± 4.7 | 31 ± 16.1$^{a, b}$ | 29 ± 16$^{A, B}$ |
| **TK 6** | Ro 19-8022 | 11.3 ± 1$^{a, b, c}$ | 54.2 ± 4$^{A, B, C}$ | | |
| | Ref. control | 2.5 ± 1.8 | 9.8 ± 3.7 | | |
| **A549** | Ro 19-8022 | | | 24.5 ± 8$^{a, b}$ | 49.3 ± 9.9$^{A, B, C}$ |
| | Ref. control | | | 3.7 ± 3.2 | 9.8 ± 6.4 |

**Table S4.** Antibodies and staining for confocal microscopy.

| | | Supplier | Dilution |
|---|---|---|---|
| Primary antibodies | Host species | | |
| Anti- pro Surfactant Protein C antibody | rabbit | ab90716 Abcam | 1:250 |
| Anti- Cd11b | mouse | AM32402PU-N-Origene | 1:200 |
| Secondary antibodies | | | |
| Donkey anti-Rabbit IgG (H+L) Highly Cross-Adsorbed Secondary Antibody, Alexa Fluor 488 | | Life technologies | 1:1000 |
| Donkey anti-Mouse IgG (H+L) highly cross-adsorbed secondary antibody, Alexa Fluor 596 | | Life technologies | 1:1000 |
| **Nuclei staining** | | | |
| DAPI | | Sigma | 1:1000 |

*4. immunofluorescence*

A representative confocal figure of the apical side (A549 /dTHP1) showed in Supplementary figure A. epithelial cells A549 stained for pro -SPC in green and dTHP1, in red, stained for cd11b1 marker for mature macrophages. We analyzed with the program Qu-Path, the number of dTHP1 respect the A549 cell line. The image is representative figure of an unexposed 3D lung model after air lifting. Here we showed how the percentage of dTHP1 viable and not damage on the apical side of the tri-culture is only the 5% of the total cells.

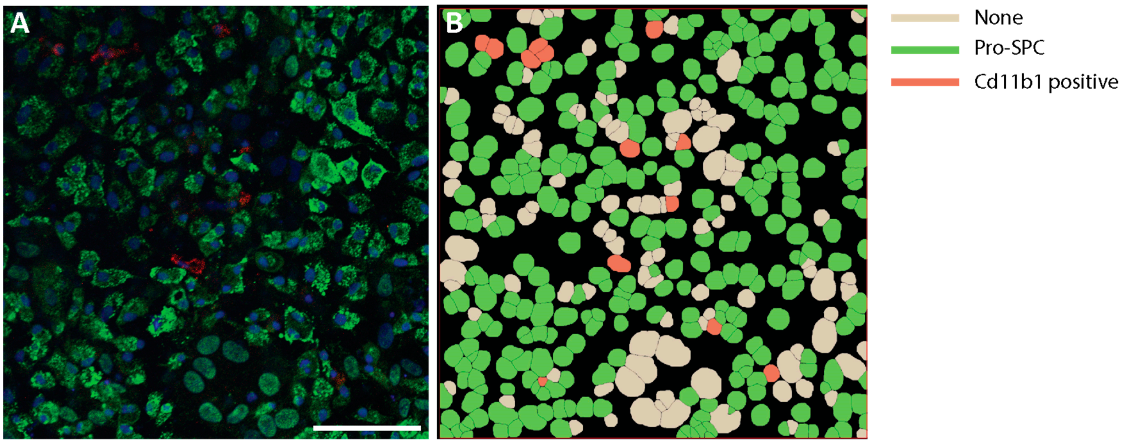


**Table S5.** Original export cell map Qu-Path*

| | Area µm$^2$ | Perimeter µm | #Total cells detected | #Cd11b1 positive | #Unclassified | #Pro-SPC positive |
|---|---|---|---|---|---|---|
| **Apical 3D lung model-unex 20x_full_original.czi** | 177018.7 | 1682.9 | Num Detections | Num Not-Pro-SPC: Cd11b1 | Num Not-Pro-SPC: Not-Cd11b1 | Num Pro-SPC: Not-Cd11b1 |
| | | | 407 | 13 | 104 | 290 |

* [4]

**Supplementary Figure 1. Confocal figure of the apical side (A549 / dTHP1) of an unexposed 3D lung triculture model after air lifting. A.** dTHP1 marked with cd11b1 (macrophages marker) in red on the top of A549 cell line. **B.** A549 epithelial cell line marked with pro surfactant protein in green (Pro- SPC). **C.** Cell Nucleai stained with DAPI in blue. **D.** Merge picture. Magnification 40X. Scale bare: 100 µm. and Table 6. Original export cell map Qu-Path. Analysis has been done on the representative immunofluorescence figure in A. Cells were differentiated by staining (colors) B. dTHP1 are results to be the 5% of total cells seeded.

## References


1. Ding, Y.; Weindl, P.; Lenz, A.G.; Mayer, P.; Krebs, T.; Schmid, O. Quartz crystal microbalances (QCM) are suitable for real-time dosimetry in nanotoxicological studies using VITROCELL(R)Cloud cell exposure systems. *Part Fibre Toxicol* **2020**, *17*, 44, doi:10.1186/s12989-020-00376-w.
2. Ding, Y.; Weindl, P.; Wimmer, C.; Mayer, P.; Krebs, T.; Schmid, O. *Characterization of the air-liquid interface cell exposure (ALICE-CLOUD) system for in-vitro toxicological studies of engineered nanomaterials (ENMs)*; 2017.
3. Camassa, L.M.; Elje, E.; Mariussen, E.; Longhin, E.M.; Dusinska, M.; Zienolddiny-Narui, S.; Rundén-Pran, E. Advanced Respiratory Models for Hazard Assessment of Nanomaterials—Performance of Mono-, Co- and Tricultures. *Nanomaterials* **2022**, *12*, doi:10.3390/nano12152609.
4. Bankhead, P.; Loughrey, M.B.; Fernández, J.A.; Dombrowski, Y.; McArt, D.G.; Dunne, P.D.; McQuaid, S.; Gray, R.T.; Murray, L.J.; Coleman, H.G.; et al. QuPath: Open source software for digital pathology image analysis. *Scientific Reports* **2017**, *7*, 16878, doi:10.1038/s41598-017-17204-5.
